# Sirt1 negatively regulates FcεRI-mediated mast cell activation through AMPK- and PTP1B-dependent processes

**DOI:** 10.1038/s41598-017-06835-3

**Published:** 2017-07-25

**Authors:** Xian Li, Youn Ju Lee, Fansi Jin, Young Na Park, Yifeng Deng, Youra Kang, Ju Hye Yang, Jae-Hoon Chang, Dong-Young Kim, Jung-Ae Kim, Young-Chae Chang, Hyun-Jeong Ko, Cheorl-Ho Kim, Makoto Murakami, Hyeun Wook Chang

**Affiliations:** 1 College of Pharmacy, Yeungnam University, 280 Daehak-Ro, Gyeongsan, Gyeongbuk 38541 Republic of Korea; 2 Department of Pharmacology, School of Medicine, Catholic University of Daegu, 33 Duryugongwon-ro 17-gil, Nam-gu, Daegu Republic of Korea; 3 Korean Medicine (KM) Application Center, Korea Institute of Oriental Medicine, 70 Cheomdan-ro, Dong-gu, Daegu, 41062 Republic of Korea; 4 Research Institute of Biomedical Engineering and Department of Medicine, Catholic University of Daegu School of Medicine, 33 Duryugongwon-ro 17-gil, Nam-gu, Daegu Republic of Korea; 5 Laboratory of Microbiology and Immunology, College of Pharmacy, Kangwon National University, 1 Kangwondaehak-gil, Chuncheon-si, Gangwon-do 24341 Republic of Korea; 6 Molecular and Cellular Glycobiology Unit, Department of Biological Sciences, SungKyunKwan University, 2066 Seobu-Ro, Suwon City, Kyunggi-Do 16419 Republic of Korea; 7 Lipid Metabolism Project, Tokyo Metropolitan Institute of Medical Science, Tokyo, 156-8506 Japan

## Abstract

Sirt1, a key regulator of metabolism and longevity, has recently been implicated in the regulation of allergic reactions, although the underlying mechanism remains unclear. Here we show that Sirt1 negatively regulates FcεRI-stimulated mast cell activation and anaphylaxis through two mutually regulated pathways involving AMP-activated protein kinase (AMPK) and protein tyrosine phosphatase 1B (PTP1B). Mast cell-specific knockout of Sirt1 dampened AMPK-dependent suppression of FcεRI signaling, thereby augmenting mast cell activation both *in vitro* and *in vivo*. Sirt1 inhibition of FcεRI signaling also involved an alternative component, PTP1B, which attenuated the inhibitory AMPK pathway and conversely enhanced the stimulatory Syk pathway, uncovering a novel role of this phosphatase. Moreover, a Sirt1 activator resveratrol stimulated the inhibitory AMPK axis, with reciprocal suppression of the stimulatory PTP1B/Syk axis, thus potently inhibiting anaphylaxis. Overall, our results provide a molecular explanation for the beneficial role of Sirt1 in allergy and underscore a potential application of Sirt1 activators as a new class of anti-allergic agents.

## Introduction

Mast cells represent a highly specialized cell population that plays a central role in allergic diseases. Crosslinking of FcεRI-bound IgE with antigen (Ag) on mast cells induces the activation of proximal FcεRI-associated Src kinases (Lyn and Fyn) and Syk, which in turn activate multiple signaling pathways including phospholipase Cγ (PLCγ), mitogen-activated protein kinases, Akt and NF-κB, leading to release of preformed mediators by degranulation and *de novo* synthesis of lipid mediators and cytokines^1, 2^. Besides these positive signaling pathways, understanding of the negative regulatory mechanisms that turn off positive signals is also of importance to gain comprehensive insights into FcεRI signaling and thereby a novel strategy for treatment of allergic diseases. Examples for such negative regulatory mechanisms involve the tyrosine phosphatases SHP-1 and SHIP in FcεRI- mediated mast cell activation^3, 4^, the Cbl family ubiquitin ligases, which facilitate degradation or internalization of the activated FcεRI signaling components^5^, and the inhibitory kinase Csk, which phosphorylates and thereby inactivates the FcεRI-proximal kinases Lyn and Fyn^1^. Additionally, we have recently shown that AMP-activated protein kinase (AMPK), which is generally known to be activated during energy insufficiency and is essential for metabolic homeostasis^6, 7^, represents a novel negative regulatory module for FcεRI signaling by altering the subcellular distribution of Fyn and ERK^8, 9^.

Sirtuin 1 (Sirt1), a ubiquitously expressed NAD^+^-dependent type III histone/protein deacetylase, deacetylates several transcriptional and related factors, thereby regulating energy metabolism, aging, senescence and inflammation^10–12^. Similar to AMPK, Sirt1 is regulated in response to energy demand and its dysregulation is associated with metabolic syndrome and inflammation^13–16^. However, the roles of Sirt1 in allergic diseases are controversial, because Sirt1 reportedly prevents or exacerbates allergic responses in distinct settings^17–21^. Some of these studies relied on the pharmacological effects of resveratrol, a red grape-derived polyphenol that has frequently been used as a Sirt1 activator. Indeed, recent studies demonstrated the therapeutic effects of resveratrol on allergic symptoms in both humans and rodents^22–27^, supporting the anti-allergic action of Sirt1. However, the roles of Sirt1in allergy have not been firmly established, since it is uncertain whether resveratrol acts on only Sirt1 or some another unknown molecule(s) to exert its actions^25–27^.

Crosstalk between Sirt1 and AMPK has attracted attention in the fields of energy homeostasis, aging and longevity^28–30^. In hepatocytes, the Sirt1 activator resveratrol activates AMPK, whereas the Sirt1 inhibitor nicotinamide suppresses both Sirt1 and AMPK^31, 32^. Resveratrol improves insulin sensitivity and mitochondrial function and extends the lifespan of obese mice through activation of Sirt1 and AMPK^29, 33^. Overexpression of Sirt1 reduces lysine acetylation of LKB1, leading to interaction with and activation of downstream AMPK^34^. Reciprocally, AMPK functions as a Sirt1 activator by increasing the level of cellular NAD^+^ or the activity of nicotinamide phosphoribosyltransferase, an NAD^+^-biosynthetic enzyme^35^. Together, Sirt1, LKB1 and AMPK are coordinately regulated to form a feed-forward cycle. Given these facts, it can be speculated that Sirt1 may have a negative regulatory role in mast cell activation through interaction with AMPK, although experimental evidence for this hypothesis has currently been lacking.

In this study, we investigated the roles of Sirt1 in mast cells using pharmacological and genetic approaches. We show that Sirt1 indeed cooperates with AMPK in mast cells, thereby dampening FcεRI signaling. Unexpectedly, the inhibitory action of Sirt1 on FcεRI signaling also relies on an alternative pathway involving protein-tyrosine phosphatase 1B (PTP1B), whose role in mast cells had been controversial. Our results show that PTP1B inhibits AMPK and activates Syk to facilitate FcεRI signaling, and these processes are counteracted by Sirt1.

## Results

### Resveratrol inhibits IgE/Ag-stimulated mast cell activation

**Figure 1:**
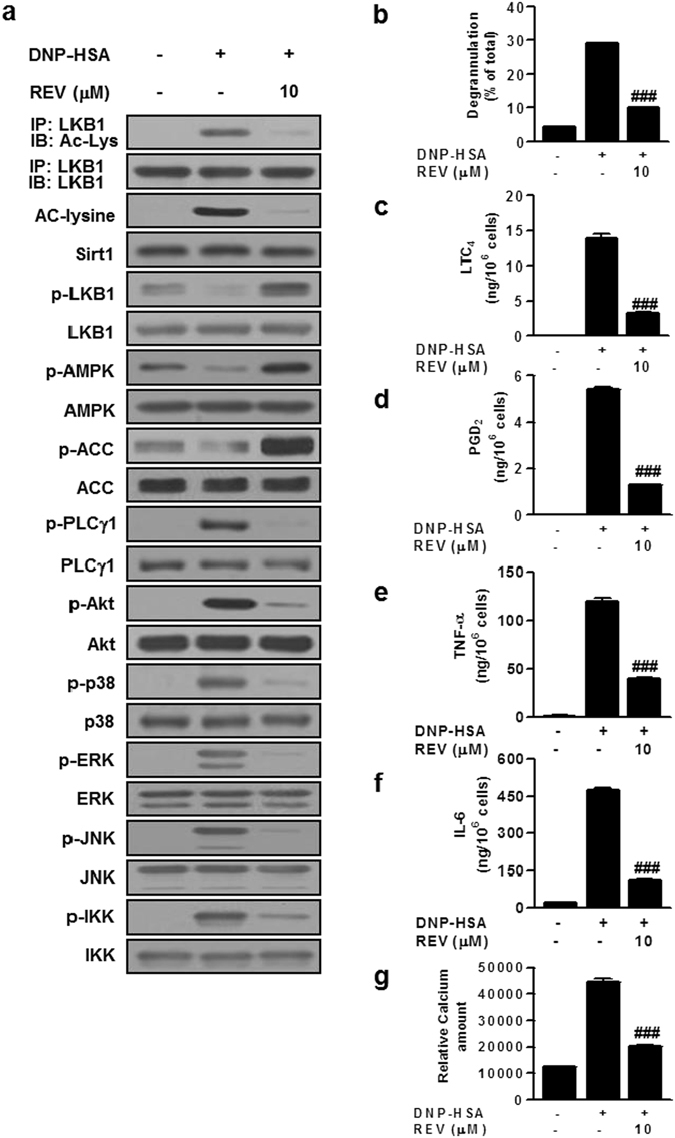
Resveratrol inhibits IgE/Ag-mediated mast cells activation. IgE-sensitized BMMCs were treated with 10 μM of resveratrol (REV) for 1 h and then stimulated with Ag. Effects of REV on acetylation or phosphorylation of signaling molecules were evaluated by immunoblotting (**a**). Releases of β-Hex (**b**), LTC_4_ (**c**) and PGD_2_ (**d**), secretion of cytokines (**e**,**f**) and influx of Ca^2+^ (**g**) were evaluated. The immunoblot data (**a**) is a representative of three independent experiments, and the values (**b–g**) indicate the means ± S.E.M. from three independent experiments with different BMMCs (^###^ *P* < 0.001 *vs*. DNP-HSA alone).

We have shown that the LKB1/AMPK axis suppresses FcεRI signaling including PLCγ1, ERK1/2, JNK and IKK without affecting Akt and p38, thereby limiting mast cell activation^8, 9^. Given that Sirt1 lies upstream of AMPK^34, 36^, we investigated the effect of resveratrol, a Sirt1 activator, on IgE/Ag-stimulated mast cell activation in the context of AMPK signaling. First, to determine the proper concentration of resveratrol for Sirt1 activation, mouse bone marrow-derived mast cells (BMMCs) sensitized with anti-dinitrophenyl (DNP) IgE were treated with various concentrations (1–100 μM) of resveratrol for 1 h prior to stimulation with DNP-human serum albumin (HSA) as an Ag. As reported previously^8, 9^, IgE/Ag stimulation resulted in a substantial decrease in constitutive phosphorylation of LKB1, AMPK, and their well-known downstream target acyl-CoA carboxylase (ACC) (Fig. 1a and Supplementary Fig. 1). Additionally, IgE/Ag stimulation increased lysine acetylation (Ac-Lys) of LKB1 (Fig. 1a). Resveratrol decreased FcεRI-induced Ac-Lys and increased phosphorylation of LKB1, AMPK and ACC in a dose-dependent manner, and its effect was evident even in unstimulated cells (Fig. 1a and Supplementary Fig. 1). Since resveratrol exerted an almost maximum effect at 10 μM, this concentration of resveratrol was used in subsequent experiments. In agreement with the negative regulatory role of AMPK in FcεRI signaling^8, 9^, resveratrol markedly decreased FcεRI-induced activation of PLCγ1, ERK1/2, JNK and IKK (Fig. 1a). In addition, resveratrol also inhibited the phosphorylation of Akt and p38 (Fig. 1a), which are not influenced by AMPK^8, 9^. Consistent with the anti-allergic action of resveratrol^22–27^ and the anti-allergic role of AMPK in FcεRI signaling^8, 9^, resveratrol attenuated IgE/Ag-mediated release of β-hexosaminidase (β-Hex), generation of the lipid mediators LTC_4_ and PGD_2_, secretion of the cytokines TNF-α and IL-6, and increase of the intracellular calcium level (Fig. 1b–g). These results raise the possibility that the deacetylation of LKB1 by Sirt1 underlies the negative regulation of mast cell activation through the LKB1/AMPK pathway and that resveratrol also affects an AMPK-independent event(s) toward inhibition of Akt and p38.

### Sirt1 suppresses mast cell activation through the LKB1/AMPK pathway

**Figure 2:**
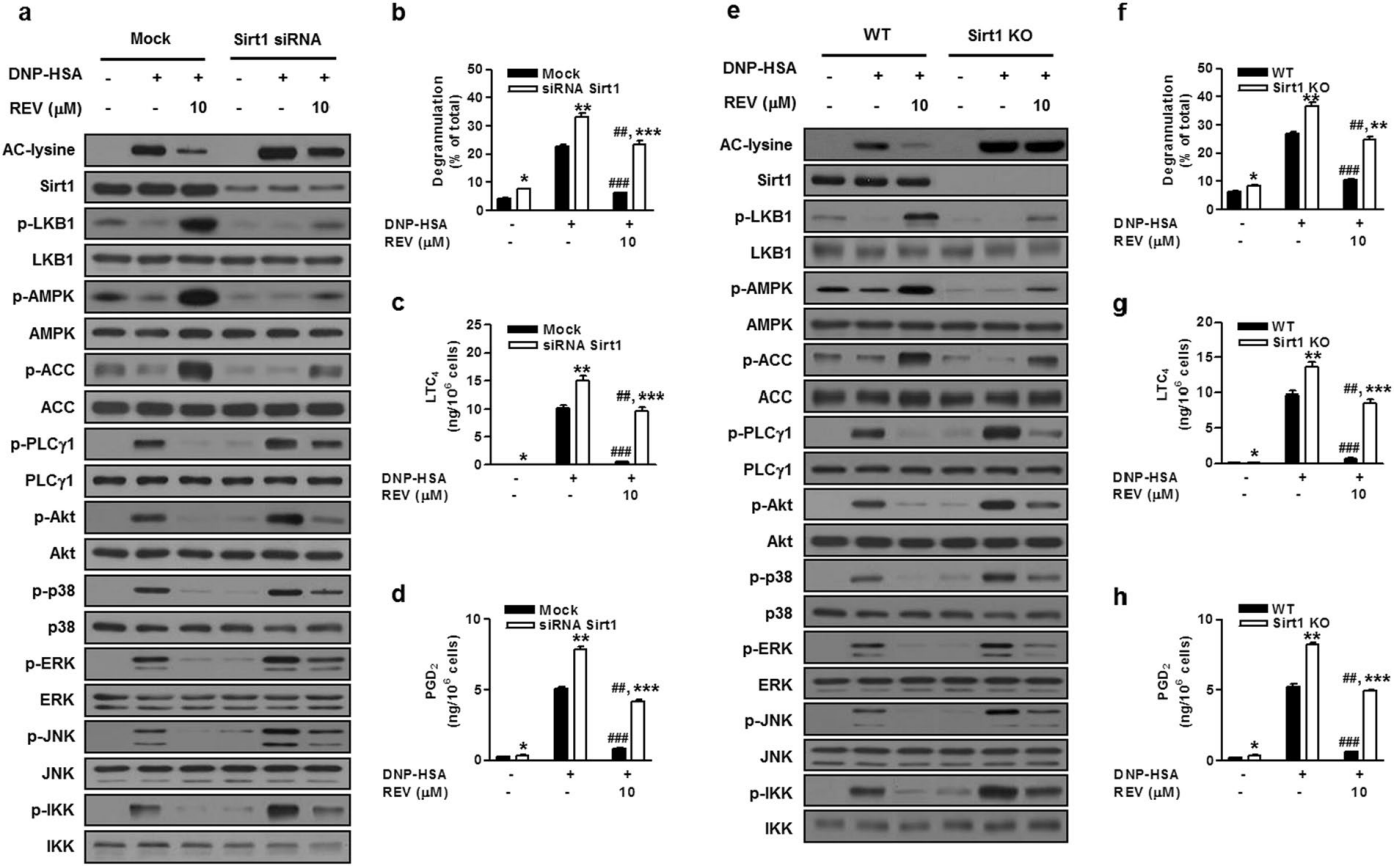
Sirt1 depletion increases IgE/Ag-induced mast cell activation. BMMCs treated with Sirt1 or control (mock) siRNA (**a**–**d**) and BMMCs from *Sirt1* ^−/−^ (KO) or WT mice (**e–h**) were stimulated with IgE/Ag in the presence or absence of REV. Acetylation or phosphorylation of signaling molecules (**a,e**) and releases of β-Hex (**b,f**), LTC_4_ (**c,g**) and PGD_2_ (**d,h**) were evaluated. The immunoblot data (**a,e**) are representative of three independent experiments, and the values (**b**–**d, f**–**h**) indicate the means ± S.E.M. from three independent experiments with different BMMCs (*P < 0.05, **P < 0.01 and ***P < 0.001 *vs*. mock or WT in each treatment; ^##^ *P* < 0.01 and ^###^ *P* < 0.001 *vs*. DNP-HSA alone in each group).

To address the role of Sirt1 further, BMMCs were transiently transfected with Sirt1-specific siRNA. Treatment of BMMCs with Sirt1 siRNA, in comparison with that with mock siRNA, greatly if not entirely abrogated the expression of Sirt1, accompanied by a substantial increase in FcεRI-induced Ac-Lys and a reduction in constitutive phosphorylation of LKB1, AMPK, and ACC (Fig. 2a and Supplementary Fig. 2), suggesting that the activation of the LKB1/AMPK/ACC axis indeed relies on Sirt1.

Additionally, Sirt1 siRNA increased IgE/Ag-induced phosphorylation of PLCγ1, ERK, JNK, and IKK as well as Akt and p38 compared with the mock control (Fig. 2a and Supplementary Fig. 2). Consistently, IgE/Ag-dependent and even spontaneous releases of β-Hex, LTC_4_ and PGD_2_ were significantly greater in Sirt1-knockdown cells than in control cells (Fig. 2b–d). These responses were counteracted by resveratrol, which decreased Ac-Lys, increased LKB1, AMPK and ACC phosphorylation, attenuated PLCγ1, Akt, ERK, JNK, p38 and IKK phosphorylation, and markedly prevented degranulation and eicosanoid generation in control cells (Fig. 2a–d and Supplementary Fig. 2). Moreover, these effects of resveratrol were also observed partially in Sirt1-silenced cells (Fig. 2a–d and Supplementary Fig. 2), probably because Sirt1 knockdown was incomplete or because resveratrol might have an additional target(s) (see below).

Conversely, adenoviral overexpression of Sirt1 (Ad-Sirt1) in BMMCs resulted in a marked reduction in IgE/Ag-induced Ac-Lys relative to mock cells (Supplementary Fig. 3a). This was accompanied by increased phosphorylation of LKB1, AMPK and ACC, decreased phosphorylation of PLCγ1, Akt, p38, ERK, JNK, and IKK, and reduced degranulation and eicosanoid generation in Ad-Sirt1-infected cells compared with control cells (Supplementary Fig. 3a–d). Adenovirus alone had no effect on the expression and activation of these signaling molecules in this experimental setting^8^. Thus, the effects of Sirt1 overexpression fully reciprocate those of its knockdown.

To obtain more solid evidence for the role of Sirt1 in mast cell signaling, we used mast cell-specific *Sirt1* ^−/−^ mice, which were obtained by crossing *Sirt1*-floxed mice^37^ with mast cell-specific *Mast-Cma1-Cre* mice^38^ on a C57BL/6 background (see Methods). Sirt1 deficiency in BMMCs completely abrogated Sirt1 protein expression and resveratrol-induced deacetylation of FcεRI-driven Ac-Lys (Fig. 2e), confirming that Sirt1 is fully responsible for this deacetylation event. Interestingly, the phosphorylation of LKB1, AMPK and ACC in *Sirt1* ^−/−^ BMMCs was still enhanced by resveratrol, although the degree of the increase was much smaller than that observed in control BMMCs, suggesting that the activation of LKB1/AMPK by resveratrol depends largely, but not solely, on Sirt1 (Fig. 2e). In agreement with Sirt1-silenced cells (Fig. 2a), notable increases in the phosphorylation of PLCγ1, Akt, p38, ERK, JNK, and IKK were seen in IgE/Ag-stimulated *Sirt1* ^−/−^ BMMCs compared with that in wild-type (WT) BMMCs (Fig. 2e). Furthermore, in accordance with the partial but not full dependence of the resveratrol effect on Sirt1, phosphorylation of PLCγ1, Akt, p38, ERK, JNK, and IKK was partly reduced by resveratrol in *Sirt1* ^−/−^ cells (Fig. 2e). Consistently, degranulation and eicosanoid generation were significantly higher in *Sirt1* ^−/−^ BMMCs than in control BMMCs, and resveratrol suppressed these responses in WT cells and even in *Sirt1* ^−/−^ cells, although the degree of the reduction in *Sirt1* ^−/−^ cells was apparently smaller than that observed in WT cells (Fig. 2f–h). These results further support the idea that resveratrol attenuates mast cell activation by driving the inhibitory Sirt1/LKB1/AMPK pathway plus another mechanism(s).

### AMPKα2 reciprocally activates Sirt1 in mast cells

**Figure 3:**
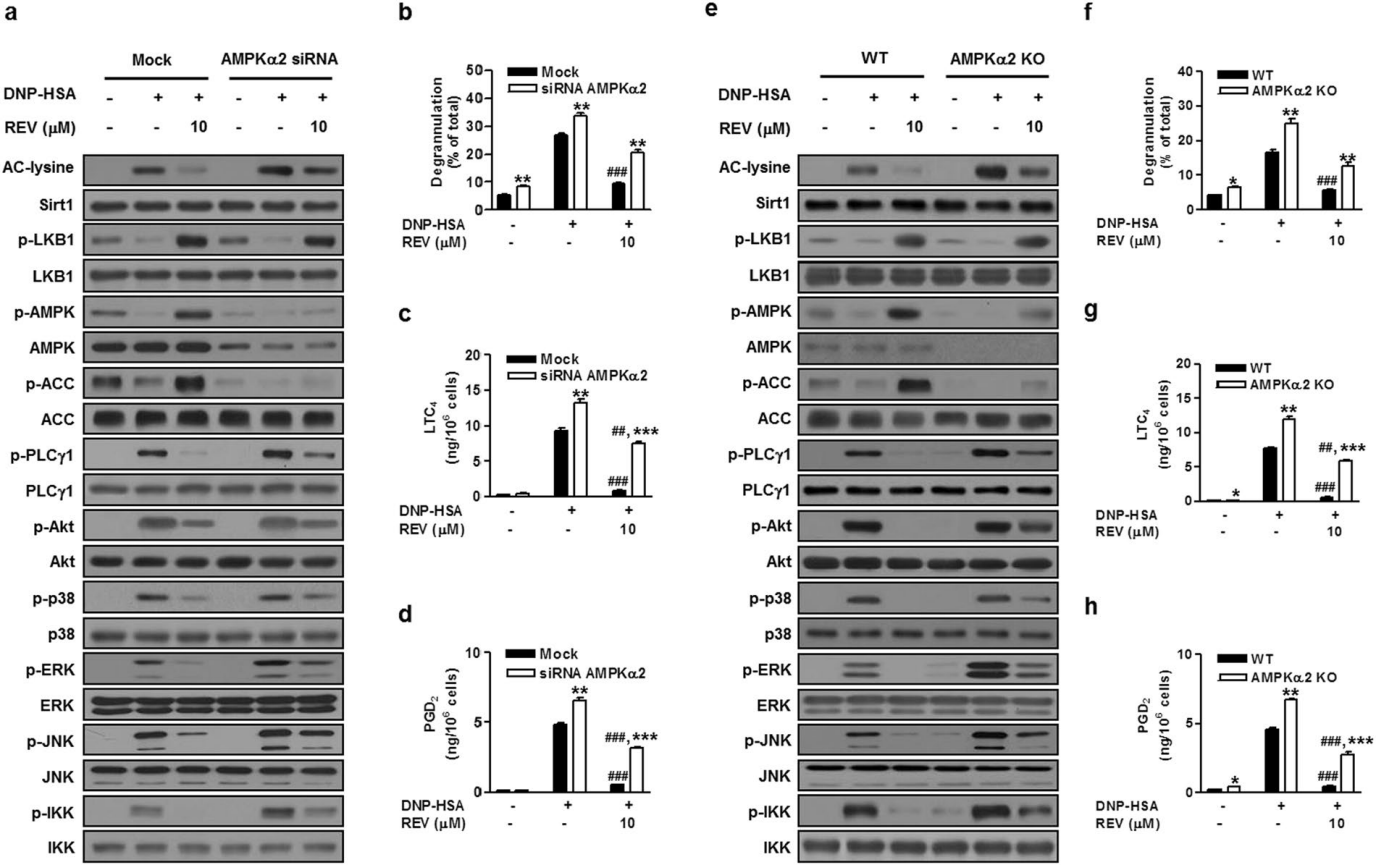
AMPKα2 depletion increases IgE/Ag-induced mast cell activation. BMMCs treated with AMPKα2 or control (mock) siRNA (**a**–**d**) and BMMCs from *AMPKα2* ^−/−^ (KO) or WT mice (**e**–**h**) were stimulated with IgE/Ag in the presence or absence of REV. Acetylation or phosphorylation of signaling molecules (**a,e**) and releases of β-Hex (**b,f**), LTC_4_ (**c,g**) and PGD_2_ (**d,h**) were evaluated. The immunoblot data (**a,e**) are representative of three independent experiments, and the values (**b**–**d,f**–**h**) indicate the means ± S.E.M. from three independent experiments with different BMMCs (*P < 0.05, **P < 0.01 and ***P < 0.001 *vs*. mock or WT in each treatment; ^##^ *P* < 0.01 and ^###^ *P* < 0.001 *vs*. DNP-HSA alone in each group).

The diverse modes of interaction between Sirt1 and AMPK have been observed under different biological conditions^11, 31^. Sirt1 is placed upstream of AMPK^34, 36^, while AMPK can act upstream of Sirt1, enhancing its deacetylase activity by modulating intracellular NAD^+^ levels^39^. Therefore, we next asked whether AMPK could reciprocally activate Sirt1 in mast cells. siRNA knockdown (Fig. 3a and Supplementary Fig. 4) or genetic knockout (Fig. 3e) of AMPKα2, a major AMPK isoform in mast cells^8^, markedly reduced the levels of its total protein and phosphorylated form, as well as constitutive phosphorylation of its target ACC, as expected. Interestingly, knockdown (Fig. 3a) or knockout (Fig. 3e) of AMPKα2 increased FcεRI-induced Ac-Lys without altering Sirt1 protein, an effect that was partially reversed by resveratrol (a slight increase in phosphorylated AMPK in resveratrol-treated *AMPK*α*2* ^−/−^ cells was likely to be ascribed to AMPKα1), suggesting that the ablation of AMPKα2 decreased the deacetylase activity of Sirt1. This action appeared to be independent of LKB1, because its phosphorylation was barely affected by AMPKα2 deficiency. As reported previously^8, 9^, FcεRI-dependent phosphorylation of PLCγ1, ERK, JNK, and IKK, but not Akt and p38, was higher in AMPKα2-silenced or -deficient cells than control cells (Fig. 3a,e and Supplementary Fig. 4). Furthermore, as in Sirt1-deleted cells (Fig. 2a,e), FcεRI-induced Ac-Lys and phosphorylation of PLCγ1, Akt, p38, ERK, JNK, and IKK were partially reduced by resveratrol in AMPKα2-deleted cells (Fig. 3a,e and Supplementary Fig. 4). Accordingly, degranulation and eicosanoid synthesis were significantly higher in AMPKα2-deleted cells than in control cells, and resveratrol significantly attenuated these responses almost completely in control cells and partially in AMPKα2-deleted cells (Fig. 3b–d,f–h). These results suggest that AMPK is required for optimal Sirt1 activation, forming a Sirt1/LKB1/AMPK feed-forward loop.

### The Sirt1/AMPK axis attenuates anaphylaxis

**Figure 4:**
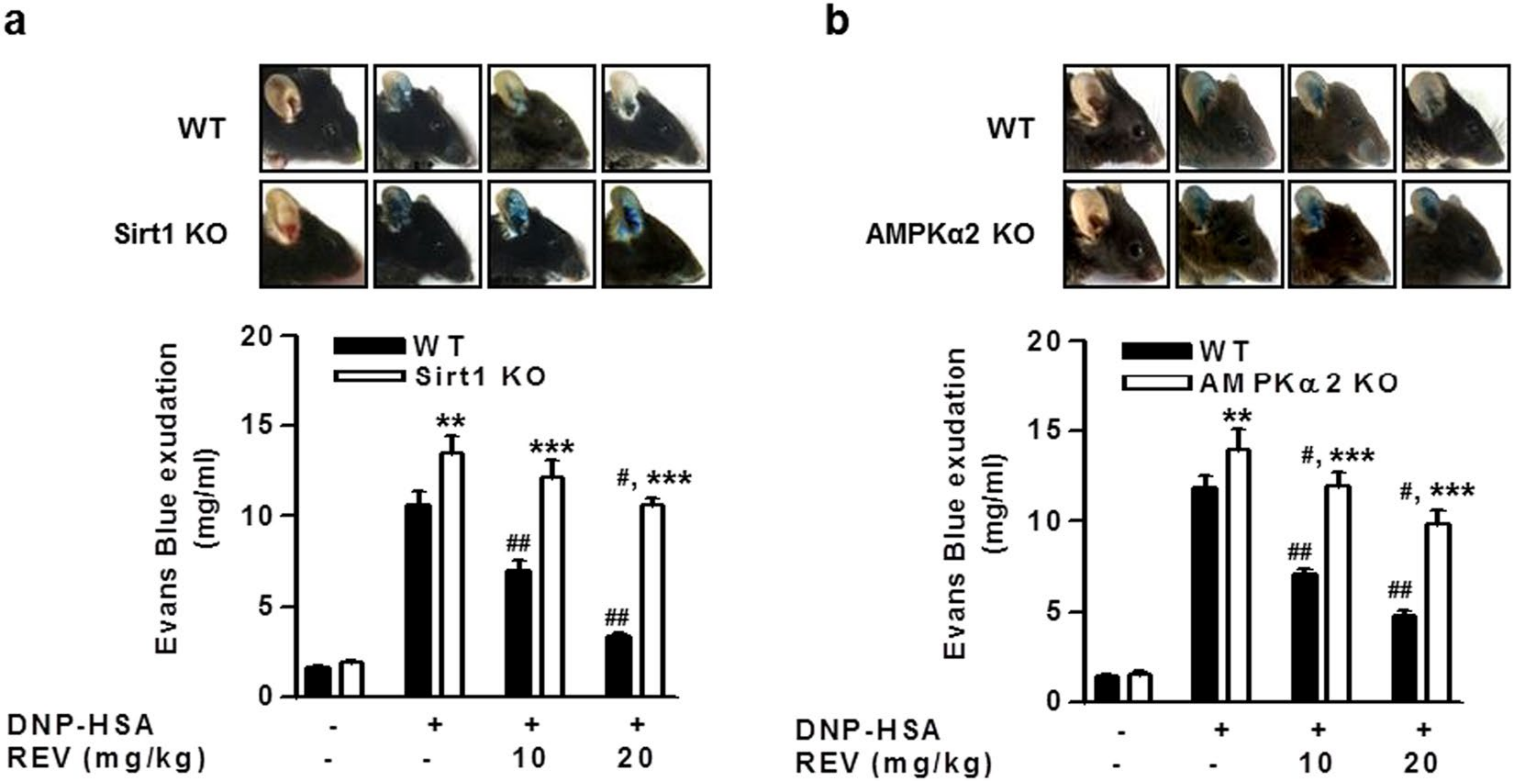
Gene ablation of *Sirt1* or *AMPK*α*2* augments mast cell-mediated anaphylaxis. IgE/Ag-induced PCA reactions in WT, mast cell-specific *Sirt1* ^−/−^ (KO) (**a**) or *AMPK*α*2* ^−/−^ (KO) (**b**) mice were evaluated in the presence or absence of REV. The amounts of evans blue exudation are presented (n = 7 mice per group; ^#^ *P* < 0.05, ^##^ *P* < 0.01 *vs*. DNP-HSA alone in either WT or KO group; **P < 0.01, ***P < 0.001 *vs*. WT for each treatment). Top panels show representative photos of ears with dye extravasation at 1 h.

To validate the pathophysiological relevance of these observations, we investigated passive cutaneous anaphylaxis (PCA) using mast cell-specific *Sirt1* ^−/−^ mice. Deficiency of Sirt1 in mast cells significantly augmented IgE/Ag-induced PCA, confirming the anti-allergic role of mast cell-intrinsic Sirt1 *in vivo* (Fig. 4a). Likewise, the PCA response was significantly greater in *AMPKα2* ^−/−^ mice than in WT mice (Fig. 4b), as reported previously^8^. Oral administration of resveratrol to WT mice reduced dye extravasation by ~40% and 70% at 10 and 20 mg/kg, respectively, whereas the same doses of resveratrol decreased it by only ~10% and 24%, respectively, in *Sirt1* ^−/−^ mice (Fig. 4a) and by only ~13% and 27%, respectively, in *AMPKα2* ^−/−^ mice (Fig. 4b). Altogether, these *in vitro* and *in vivo* results indicate that the Sirt1/LKB1/AMPK circuit shuts off FcεRI-mediated mast cell activation and that the effect of resveratrol involves both Sirt1/LKB1/AMPK-dependent and -independent mechanisms.

### Sirt1 mutually regulates the inhibitory LKB1/AMPK and stimulatory PTP1B/Syk axes

**Figure 5:**
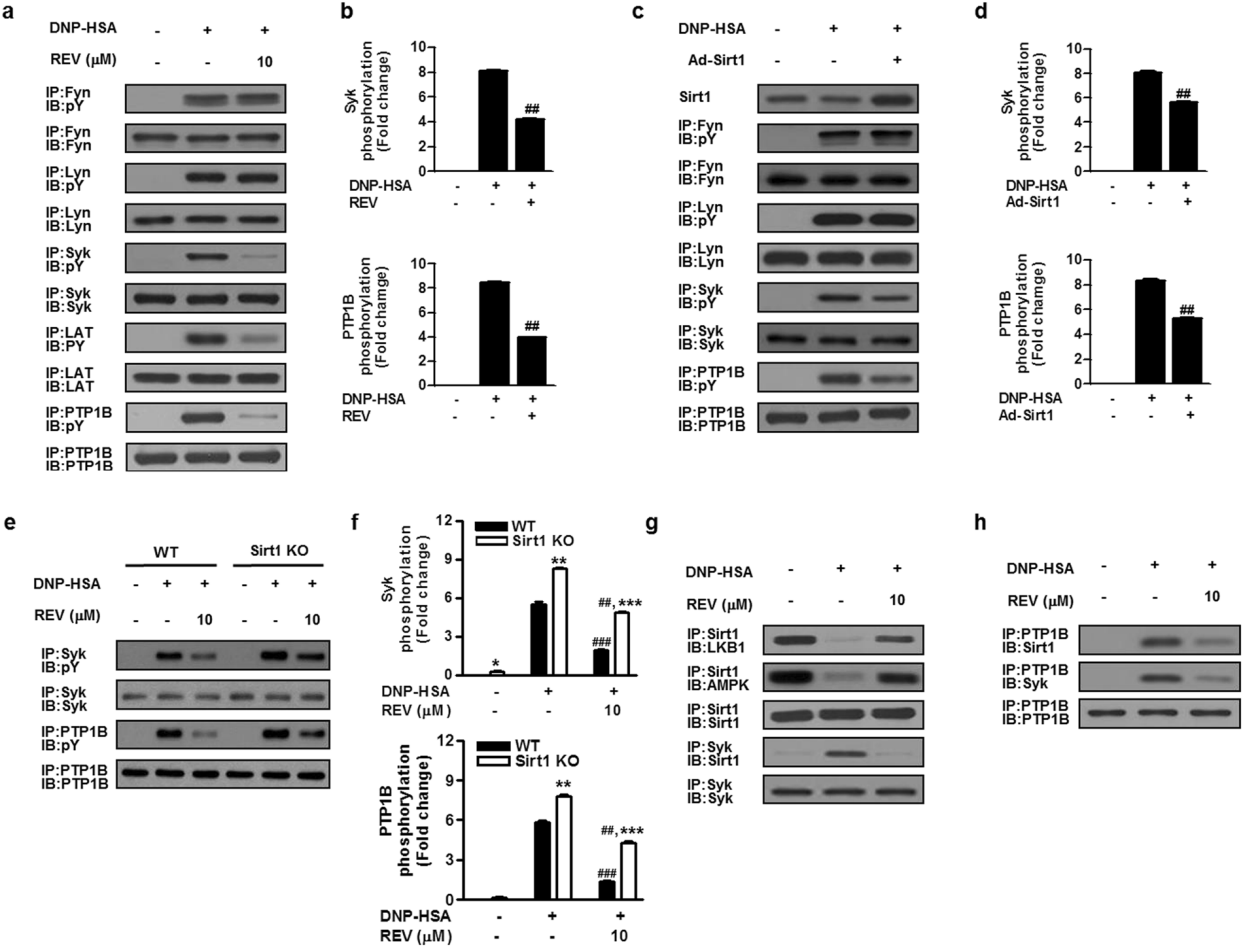
Resveratrol or Sirt1 decreases the phosphorylation of Syk and PTP1B. Effects of REV (**a,b**), Sirt1 overexpression (Ad-Sirt1) (**c,d**) or Sirt1 knockout (KO) (**e,f)** on the phosphorylation of signaling molecules were evaluated. The relative ratios of phosphorylated to total Syk and PTP1B were determined by scanning densitometry (**b,d,f**). (**g,h**) Effects of REV on protein interactions among Sirt1, LKB1, AMPK, Syk and PTP1B. The immunobot data (**a,c,e,g,h**) are representative of three independent experiments, and the values (**b,d,f**) indicate the means ± S.E.M. from three independent experiments with different BMMCs (^##^ *P* < 0.01 *vs*. DNP-HSA alone; **P < 0.01, ***P < 0.001 *vs*. WT in each treatment).

Our results suggest that resveratrol has an additional target(s) other than the Sirt1/LKB1/AMPK axis. Therefore, we examined whether resveratrol could affect the phosphorylation of FcεRI-proximal tyrosine kinases, Lyn, Fyn and Syk. Resveratrol attenuated IgE/Ag-induced phosphorylation of Syk and its target adaptor LAT, without affecting that of Lyn and Fyn (Fig. 5a,b). The effect of resveratrol was recapitulated by adenoviral Sirt1 overexpression, which reduced Syk, but not Lyn and Fyn, phosphorylation (Fig. 5c,d). Furthermore, IgE/Ag-induced Syk phosphorylation was greater in *Sirt1* ^−/−^ cells than in WT cells, and resveratrol reversed it markedly in WT cells and partially in *Sirt1* ^−/−^ cells (Fig. 5e,f). Because Syk is the primary tyrosine kinase essential for FcεRI signaling^40, 41^, it appeared that the attenuation of all branches of FcεRI signaling by Sirt1 could be accounted for, at least in part, by the inhibition of Syk independently of AMPK, and that the inhibition of Syk by resveratrol relies on Sirt1-dependent and -independent mechanisms.

Notably, Sirt1 was constitutively associated with LKB1/AMPK in unstimulated BMMCs, whereas it dissociated from LKB1/AMPK and instead interacted with Syk in IgE/Ag-stimulated BMMCs (Fig. 5g). Moreover, these interactions were reversed by resveratrol, which enhanced the association of Sirt1/LKB1/AMPK and weakened that of Sirt1/Syk in IgE/Ag-treated cells (Fig. 5g). Thus, the interactions of Sirt1 with LKB1/AMPK and Syk in mast cells are mutual, being reciprocally regulated by IgE/Ag stimulation, and both processes are sensitive to resveratrol.

To search for an additional Sirt1-regulated component, we were interested in PTP1B, because it has been reported that resveratrol activation or Sirt1 overexpression improves insulin signaling through regulation of PTP1B^42^. While SHP-1 is a well-known protein tyrosine phosphatase that negatively regulates FcεRI signaling^3, 43^, the role of PTP1B in mast cell activation is currently obscure^44^. We found that PTP1B phosphorylation was robustly increased following IgE/Ag stimulation, which was reversed by resveratrol (Fig. 5a,b) or Sirt1 overexpression (Fig. 5c,d) and increased by Sirt1 knockout (Fig. 5e,f). Furthermore, PTP1B was associated with Syk and Sirt1 in activated but not in resting cells, and resveratrol markedly reduced their interactions (Fig. 5h). Thus, Syk and PTP1B undergo coordinated regulation upon FcεRI signaling, being associated with Sirt1 in a resveratrol-sensitive fashion. Under the same conditions, neither Sirt1, LKB1, AMPK, Syk nor PTP1B was precipitated with control IgG antibody (Supplementary Fig. 5).

**Figure 6:**
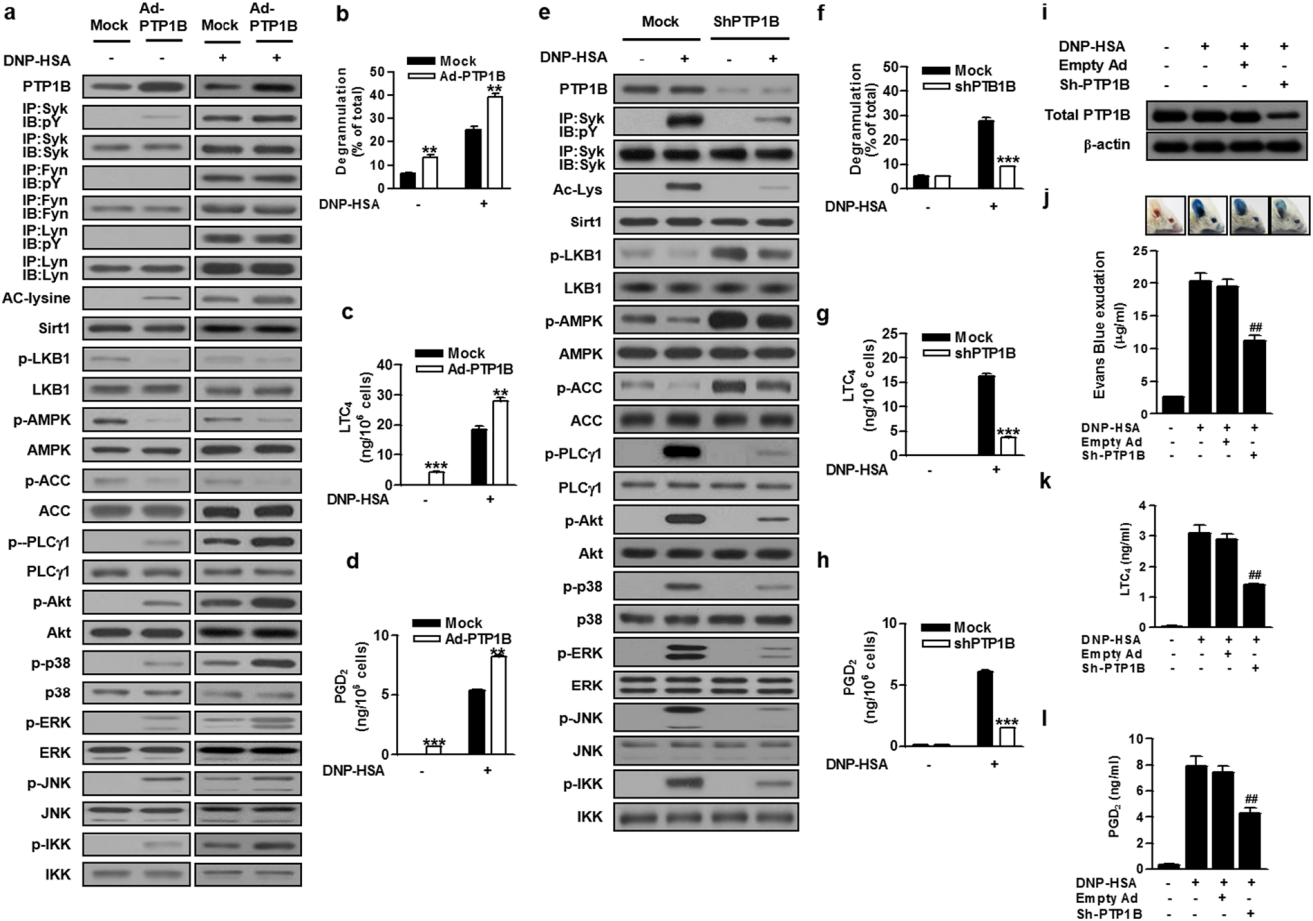
PTP1B enhances Syk signaling and decreases AMPK signalling. BMMCs transfected with adenovirus carrying PTP1B (Ad-PTP1B) or empty adenoviral vector (mock) (**a–d**) and PTP1B or mock shRNA (**e–h**) were stimulated with IgE/Ag. Acetylation or phosphorylation of signaling molecules was evaluated by immunoblotting (**a,e**). The releases of β-Hex (**b,f**), LTC_4_ (**c,g**) and PGD_2_ (**d,h**) were evaluated. (**i–l)** Mouse ears were intradermally injected with control adenovirus (empty Ad) or adenovirus carrying PTP1B shRNA (sh-PTP1B). After PCA reaction, the expression of PTP1B protein (**i**), extravasation of Evans blue dye (**j**) and the levels of serum LTC_4_ (**k**) and PGD_2_ (**l**) were evaluated (n = 6 mice per group; ^##^ *P* < 0.01 *vs*. DNP-HSA alone). Top panels in (**j**) show representative photos of ears with dye extravasation at 1 h. The immunoblot data (A, E, I) are representative of three independent experiments, and the values (**b–d,f–h,j–l**) indicate the means ± S.E.M. from three independent experiments with different BMMCs (**P < 0.01 and ***P < 0.001 *vs*. mock in each treatment, and ^##^ *P* < 0.01 and ^###^ *P* < 0.001 *vs*. DNP-HSA alone in mock or knockdown group).

To delineate the role of PTP1B in mast cell activation, we overexpressed or silenced PTP1B in BMMCs. Adenoviral overexpression of PTP1B (Ad-PTP1B) increased Ac-Lys and attenuated the phosphorylation of LKB1, AMPK, and ACC in IgE/Ag-stimulated cells and even in resting cells (Fig. 6a and Supplementary Fig. 6), suggesting that PTP1B suppresses LKB1 deacetylation by Sirt1 and thereby LKB1/AMPK activation, likely through facilitating the dissociation of Sirt1 from LKB1/AMPK (Fig. 5g,h). Strikingly, PTP1B overexpression resulted in augmented IgE/Ag-induced and even spontaneous phosphorylation of Syk and its downstream molecules PLCγ1, Akt, p38, ERK, JNK, and IKK, with no effect on phosphorylation of Lyn or Fyn (Fig. 6a and Supplementary Fig. 6). Consequently, Ad-PTP1B enhanced IgE/Ag-induced or even spontaneous release of β-Hex, LTC_4_, and PGD_2_ (Fig. 6b–d). The positive regulatory role of PTP1B in mast cell activation was further confirmed by PTP1B knockdown using a specific shRNA, which decreased FcεRI-induced Ac-Lys, enhanced constitutive phosphorylation of LKB1, AMPK, and ACC, and prevented FcεRI-induced phosphorylation of Syk as well as downstream PLCγ1, Akt, p38, ERK, JNK and IKK (Fig. 6e and Supplementary Fig. 7). Accordingly, IgE/Ag-induced degranulation and eicosanoid generation were significantly ameliorated by PTP1B knockdown (Fig. 6f–h).

To assess whether PTP1B could be involved in the regulation of mast cell-mediated anaphylaxis, we investigated IgE/Ag-induced PCA reaction in mice with PTP1B knockdown. Intradermal administration of adenovirus bearing PTP1B shRNA, but not control adenovirus, into mice decreased the expression of PTP1B protein (Fig. 6i). Consistent with the marked decreases of LTC_4_ and PGD_2_ generation in PTP1B-silenced BMMCs (Fig. 6g,h), the *in vivo* knockdown of PTP1B significantly reduced PCA-induced dye extravasation by ~45% (Fig. 6J), with parallel decreases of serum LTC_4_ and PGD_2_ levels by ~45% (Fig. 6k,l), compared with control adenovirus-treated mice, in which PCA reaction was unaffected. These data suggest that PTP1B plays a positive role in IgE/Ag-mast cell activation and associated anaphylaxis *in vivo*.

Taken together, PTP1B mutually regulates the LKB1/AMPK and Syk pathways in negative and positive ways, respectively, leading to augmented FcεRI-dependent mast cell activation. Moreover, Sirt1, a resveratrol target, acts as a regulator to counterbalance these two pathways.

## Discussion

Crosstalk between Sirt1 and AMPK, as evidenced by the findings of their shared common targets and their reciprocal regulation in diverse cellular responses^10, 11, 13, 45^, raises the possibility that Sirt1, in cooperation with AMPK^8, 9^, may negatively regulate mast cell activation. Although several studies suggest the protective effect of resveratrol against allergic responses^22–27^, the precise role of Sirt1 in FcεRI-mediated mast cell activation has not been firmly established. In this study, we have shown that resveratrol blunts FcεRI signaling partly through the activation of Sirt1, which is linked to the LKB1/AMPK pathway. FcεRI crosslinking increases Ac-Lys of LKB1, which is deacetylated by Sirt1. This deacetylation leads to increased phosphorylation and activation of LKB1/AMPK, thereby shutting down mast cell activation. Moreover, Sirt1 inhibits the activation of Syk, a central regulator of FcεRI signaling^40, 41^, and this process involves an additional player, PTP1B. Thus, activation of the inhibitory LKB1/AMPK axis and inhibition of the stimulatory PTP1B/Syk axis may underlie the sequestration of FcεRI-driven mast cell activation by Sirt1. The amelioration of effector functions in mouse BMMCs by resveratrol is consistent with a very recent study using human skin mast cells^27^, with a few minor differences (*e.g*. TNFα secretion) probably due to differences in animal species, anatomical sources, or experimental conditions. Nonetheless, our present study is the first to demonstrate the mechanistic actions of Sirt1 in mast cells.

We provide several lines of evidence that Sirt1 and AMPK require each other for their optimal activation, forming a feed-forward cycle. Ablation of Sirt1 by siRNA knockdown or genetic deletion reduced the activation of AMPK, and *vice versa*. Consistently, overexpression of Sirt1 increased the activation of AMPK. Furthermore, Sirt1 overexpression mimicked the effects of resveratrol, whereas its down-regulation attenuated the sensitivity to resveratrol and increased mast cell activation. Likewise, AMPK knockdown decreased Sirt1 activity and increased FcεRI-mediated signaling. Importantly, both mast cell-specific *Sirt1* ^−/−^ mice and *AMPK*α*2* ^−/−^ mice displayed increased susceptibility to anaphylaxis, with a diminished anti-allergic effect of resveratrol, implying the physiological relevance of our observations. Although there are contrasting reports of the offensive and protective roles of Sirt1 in mouse asthma models^19–21^, our results support the anti-allergic function of Sirt1. Presumably, the pro- or anti-inflammatory roles of Sirt1 may depend on the cell types involved, which may differ among experimental settings.

In light of the finding that Sirt1 activation by resveratrol regulates PTP1B leading to enhanced insulin sensitivity^42^, we herein show, for the first time, that PTP1B has a positive role in FcεRI signaling in two ways. On one hand, PTP1B is activated and associated with Syk toward increased FcεRI signaling. On the other hand, it counteracts the inhibitory AMPK axis by sequestering Sirt1 from the LKB1/AMPK complex. Sirt1 mutually interacts with PTP1B/Syk and LKB1/AMPK toward inhibition and activation of the positive and negative pathways, respectively, thereby dampening mast cell activation. Although it has been reported that Sirt1 downregulates PTP1B expression in insulin signaling^42^, our results do not agree with this observation, but rather support the idea that Sirt1 inhibits PTP1B activation without affecting its expression. While the activation of LKB1/AMPK by Sirt1 involves deacetylation of LKB1, it remains to be determined whether the deacetylation of PTP1B, Syk, or another unknown component(s) underlies the Sirt1 inhibition of PTP1B/Syk.

Syk has multiple tyrosine phosphorylation sites, which are involve in positive or negative regulation of Syk signaling^46^. Of these sites, phosphorylation of Tyr^317^ in the linker region of Syk not only suppresses its kinase activity, but also provides a binding site for the ubiquitin ligase Cbl, which promotes the degradation of Syk^46, 47^. Co-expression of Cbl with Syk decreases the autophosphorylated pool of Syk, eventually hindering Syk signaling^5^. Reminiscent of this, beyond the contribution of PTP1B to attenuation of insulin signaling^48, 49^, PTP1B plays a positive role in activation of Src tyrosine kinase, where it dephosphorylates the negative regulatory domain of Src and thereby activates it^50^. We therefore speculated that PTP1B may be responsible for the dephosphorylation of Tyr^317^ of Syk, thereby increasing its activity or stability. As opposed to our speculation, however, the phosphorylation of Tyr^317^ was increased (rather than decreased) by FcεRI-driven mast cell activation and was conterregulated by resveratrol, without a change in the protein level of Syk (data not shown), arguing against the hypothesis that Tyr^317^ is a dephosphorylation site for PTP1B. Additionally, the other candidate Tyr residues, Tyr^352^ and Tyr^525^, of Syk were also phosphorylated, rather than dephosphorylated, following IgE/Ag-stimulation, without being affected by resveratrol (data not shown). These results suggest that PTP1B regulates Syk activation either directly by targeting phosphotyrosine residue(s) other than Tyr^317^, Tyr^352^ and Tyr^525^ of Syk or indirectly by dephosphorylating other signaling molecule(s) involved in Syk activation. Therefore, it should be interesting to identify the target site(s) of PTP1B during mast cell activation in the future study to fully understand the underlying mechanisms for Syk regulation by PTP1B.

The roles of PTP1B in exacerbation or amelioration of inflammation are controversial. Reminiscent of our present study, PTP1B has been reported to contribute to exacerbation of neuroinflammation^51^. In contrast, a study using *PTP1B* ^−/−^ mice has shown that PTP1B plays a role in amelioration of allergen-induced airway inflammation and leukocyte trafficking^52^. These conflicting outcomes may be because the asthmatic model in the latter study depends on eosinophils and T cells rather than mast cells. In addition, unlike our present results as evaluated by transient by PTP1B knockdown or overexpression, a recent study employing PTP1B knockout has led to the conclusion that PTP1B has a negligible role in mast cell activation^44^. Although the reason for this discrepancy is unclear, similar situations have also been reported for other FcεRI signaling molecules such as Fyn and Lyn^8, 53, 54^. This difference could be because permanent knockout might have some developmental changes that ensure compensatory adaptation, while knockdown is an acute effect devoid of such adaptation. Alternatively, PTP1B might affect other signaling pathways which could vary according to experimental conditions or cellular sources.

Overall, our present findings are summarized in Supplementary Figure 8. Under unstimulated conditions, Sirt1, LKB1 and AMPK form a trimeric complex, putting a brake on mast cell activation, whereas PTP1B and Syk do not interact with each other. FcεRI crosslinking induces the interaction and activation of PTP1B and Syk toward mast cell activation, at which time the PTP1B/Syk complex allows dissociation of Sirt1 from LKB1/AMPK, thus attenuating the AMPK-driven negative regulatory module. Sirt1 in turn interacts with and inhibits PTP1B/Syk in order not to allow hyperactivation of FcεRI signaling. Sirt1 activator, resveratrol activates both arms of Sirt1 actions, leading to robust activation of the inhibitory LKB1/AMPK axis and inhibition of the stimulatory PTP1B/Syk axis. Nonetheless, the finding that resveratrol still partially attenuated the activation of *Sirt1* ^−/−^ mast cells suggests that the resveratrol effect also relies in part on a Sirt1-independent mechanism, which remains to be elucidated. Considering that mast cell activation can be suppressed by anti-oxidants^3, 55^ the anti-oxidant moiety of polyphenol might account for the Sirt1-independent action of resveratrol. Alternatively, other sirtuin members may compensate for Sirt1 in mast cells.

Understanding of the mechanisms underlying allergic reactions is still incomplete. Besides therapeutics that have been clinically used to date, an alternative approach for the treatment of allergic diseases is desired. In this context, a strategy that activates Sirt1 may have a novel therapeutic potential to treat allergic diseases.

## Methods

### Mice

Balb/cJ and C57BL/6J mice were obtained from Samtako, INC. *AMPK*α*2* ^−/−^ mice on the C57BL/6J background were reported previously^56^.

*Mast-Cma1-Cre* and *Sirt1*-floxed mice were purchased from Jackson Laboratories (Bar Harbor, ME, USA). *Mast-Cma1-Cre* mice were backcrossed for 7 to 9 generations to the C57BL/6J background and then crossed with *Sirt1*-floxed mice (C57BL/6J background) to produce age-matched *Sirt1* ^*+/+*^ *Mast-Cma1-Cre* and *Sirt1* ^*fl/fl*^ *Mast-Cma1-Cre* (termed *Sirt1* ^−/−^ hereafter) mice. All animal experiments were performed in accordance with protocols approved by the Institutional Animal Care and Use Committee of the Yeungnam University^8^.

### Preparation and activation of mouse BMMCs

BMMCs were isolated from 6~7-wk-old male Balb/cJ or C57BL/6 mice, as described previously^8^. Briefly, BMMCs were cultured in RPMI 1640 medium containing 10% (v/v) FBS, 100 U/ml penicillin (Thermo Fisher Scientific), 10 mM HEPES (Sigma-Aldrich), 100 μM MEM non-essential amino acid solution (Invitrogen) and 20% (w/v) PWM-SCM (pokeweed mitogen-spleen cell conditioned medium) as a source of IL-3. For cell stimulation, 1 × 10^6^ cells/ml were sensitized with 500 ng/ml mouse anti-DNP IgE (Sigma-Aldrich) overnight and then stimulated with 100 ng/ml DNP-HAS (Sigma-Aldrich) typically for 15 min at 37 °C. Intracellular Ca^2+^ levels at 5 min, releases of β-Hex (a marker of mast cell degranulation) and eicosanoids (LTC_4_ and PGD_2_) at 15 min, and production of cytokines (IL-6 and TNF-α) at 6 h were evaluated as described previously^8^. PGD_2_, LTC_4_, IL-6 and TNF-α were quantified using respective immunoassay kits for eicosanoids (Cayman Chemicals) and for cytokines (R&D Systems). When the effects of resveratrol (Sigma-Aldrich) were examined, it was dissolved in DMSO and added 1 h prior to the addition of Ag, with DMSO at a final concentration of 0.1% (v/v) as a vehicle control in all cases.

### Immunoprecipitation and immunoblotting

Immunoprecipitation and immunoblotting were performed as described previously^8^. Briefly, cells were washed twice with ice-cold PBS and lysed in SDS-sample buffer containing 1% (v/v) NP-40, 50 mM Tris (pH 8.0), 150 mM NaCl, 1 mM EDTA, 1 mM sodium orthovanadate, 1 mM dithiothreitol, 1 mM phenylmethylsulfonyl fluoride, 2 μg/ml aprotinin, 2 μg/ml leupeptin, and 1 μg/ml pepstatin A for 30 min on ice. Lysates were centrifuged at 14,000 *g* for 20 min at 4 °C and resulting supernatants were subjected to immunoblotting. For immnoprecipitation, cell lysates were prepared in modified lysis buffer [0.1% NP-40, 50 mM HEPES (pH 7.0), 250 mM NaCl, 5 mM EDTA, 1 mM phenylmethylsulfonyl fluoride, and 0.5 mM dithiothreitol]. Total cell lysates (1 mg protein equivalent) were incubated with various antibodies for 2 h at 4 °C and the immune complexes were precipitated with 20 μl of protein A-Sepharose. The precipitates were then washed three times with ice-cold lysis buffer. The precipitates or total cell lysates were subjected to SDS-PAGE and immunoblotted with corresponding antibodies.

### Antibodies

Antibodies against phosphorylated forms of LKB1(Ser428), AMPKα2 (Thr172), ACC (Ser79), PLCγ1 (Tyr783), Akt (Ser473), p38, ERK1/2, JNK, IKKα/β and those against LKB1, AMPKα, ACC, Akt, p38, ERK1/2, JNK and Sirt1 were from Cell Signaling Technology. Antibodies against IKKα/β, PLCγ1, LAT, Syk, Fyn, Lyn, and β-actin were from Santa Cruz Biotechnology. Anti-phosphotyrosine (pY) and –acetyl-lysine (Ac-Lys) antibodies were from Millipore, anti-PTP1B antibody was from ECM Biosciences, and anti-AMPKα2 antibody was from Abcam. Rabbit IgG was from Gen Tex.

### Gene silencing

Knockdown experiments were carried out as described previously^8^. Mouse AMPKα2 siRNA and Sirt1 siRNA in the SMARTpool were obtained from Dharmacon, and non-specific siRNA and mouse PTP1B shRNA were obtained from Santa Cruz Biotechnology. BMMCs were cultured for 16 h in serum-free medium and transfected with a DharmaFECT transfection reagent (Dharmacon) containing siRNA (100 nM per well) or shRNA lentiviral particles (5 × 10^4^ IFU per well) according to the manufacturer’s protocol in 12-well plates. After 24 h, BMMCs were sensitized with IgE in the presence or absence of resveratrol and then stimulated with DNP-HSA as above.

### Adenoviral transfection

BMMCs were infected with adenovirus carrying Sirt1 (Ad-Sirt1) or PTP1B (Ad-PTP1B) (Vector Biolabs) at 100 MOI (multiplicity of infection) according to the manufacturer’s protocol. After 24 h, medium was replaced with fresh RPMI1640 and then the cells were stimulated with IgE/DNP-HSA as described above.

### IgE/Ag-mediated PCA

PCA was carried out as described previously^8^. Briefly, 80 ng of mouse anti-DNP IgE was intradermally injected into ears of 7-wk-old male mice. After 24 h, mice were challenged intravenously with 60 ng of DNP-HSA containing Evans blue. As required for experiments, oral administration of resveratrol was given 1 h before PCA. After 1 h, Evans blue was extracted with formamide at 63 °C overnight and quantified by absorbance at 630 nm. In another set of experiments, 10^9^ PFU of control adenovirus (provided by Dr. H.J. Ko, College of Pharmacy, Kangwon National University) or 1~2 × 10^9^ PFU of adenovirus bearing shRNA for PTP1B (Ad-PTP1B-shRNA) (Vector Biolabs) was intradermally administered into mice ears. After 6 h, mice were sensitized with anti-DNP IgE for 24 h and then challenged with DNP-HSA as described above. Blood was collected by cardiac puncture at 1 h after Ag challenge to determine serum LTC_4_ and PGD_2_ levels as described above. Preparation of immunoblot samples from ear tissues were carried out as described previously^57^.

### Statistical analysis

Data calculation and statistical analysis were performed using GraphPad Prism 3.0 software. The statistical significance of differences between two groups was determined with unpaired Student’s *t* test and multiple comparisons were analyzed using one-way ANOVA. All data are presented as means ± S.E.M. Differences were considered statistically significant at *P* < 0.05.

## Electronic supplementary material

Supplementary information
