# Author Correction: Sirt1 negatively regulates FcεRI-mediated mast cell activation through AMPK- and PTP1B-dependent processes

**DOI:** 10.1038/s41598-020-60641-y

**Published:** 2020-02-21

**Authors:** Xian Li, Youn Ju Lee, Fansi Jin, Young Na Park, Yifeng Deng, Youra Kang, Ju Hye Yang, Jae-Hoon Chang, Dong-Young Kim, Jung-Ae Kim, Young-Chae Chang, Hyun-Jeong Ko, Cheorl-Ho Kim, Makoto Murakami, Hyeun Wook Chang

**Affiliations:** 1 College of Pharmacy, Yeungnam University, 280 Daehak-Ro, Gyeongsan, Gyeongbuk, 38541 Republic of Korea; 2 Department of Pharmacology, School of Medicine, Catholic University of Daegu, 33 Duryugongwon-ro 17-gil, Nam-gu, Daegu Republic of Korea; 3 Korean Medicine (KM) Application Center, Korea Institute of Oriental Medicine, 70 Cheomdan-ro, Dong-gu, Daegu 41062 Republic of Korea; 4 Research Institute of Biomedical Engineering and Department of Medicine, Catholic University of Daegu School of Medicine, 33 Duryugongwon-ro 17-gil, Nam-gu, Daegu Republic of Korea; 5 Laboratory of Microbiology and Immunology, College of Pharmacy, Kangwon National University, 1 Kangwondaehak-gil, Chuncheon-si, Gangwon-do 24341 Republic of Korea; 6 Molecular and Cellular Glycobiology Unit, Department of Biological Sciences, SungKyunKwan University, 2066 Seobu-Ro, Suwon City, Kyunggi-Do 16419 Republic of Korea; 7 Lipid Metabolism Project, Tokyo Metropolitan Institute of Medical Science, Tokyo, 156-8506 Japan

Correction to: *Scientific Reports* 10.1038/s41598-017-06835-3, published online 25 July 2017

This Article contains an error in Figure 4a and Figure 4b, where the units for ‘Evans Blue exudation’ were incorrectly given as ‘mg/ml’. The correct Figure 4 appears below as Figure 1.

**Figure 1:**
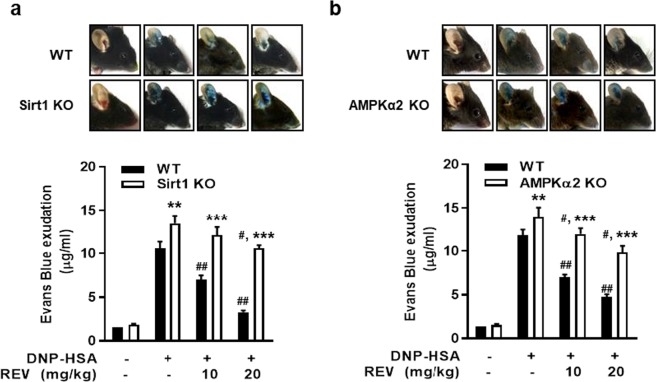
.

