# Supplementary material for: Sirt1 negatively regulates FcεRI-mediated mast cell activation through AMPK- and PTP1B-dependent processes
Source: Sci Rep. 2017 Jul 25;7:6444. doi: 10.1038/s41598-017-06835-3 (PMC5527079; doi:10.1038/s41598-017-06835-3)
Supplement: Supplementary file 1 — Supplementary information [file 41598_2017_6835_MOESM1_ESM.pdf]

Supplementary information

Sirt1 negatively regulates FcεRI-mediated mast cell activation through AMPK- and PTP1B-dependent processes

Xian Li[1#], Youn Ju Lee[2#], Fansi Jin[1], Young Na Park[1], Yifeng Deng[1], Youra Kang[1], Ju Hye Yang[3], Jae-Hoon Chang[1], Dong-Young Kim[1], Jung-Ae Kim[1], Young-Chae Chang[4], Hyun-Jeong Ko[5], Cheorl-Ho Kim[6*], Makoto Murakami[7], and Hyeun Wook Chang[1*]

[1]College of Pharmacy, Yeungnam University, 280 Daehak-Ro, Gyeongsan, Gyeongbuk 38541, Republic of Korea

[2]Department of Pharmacology, School of Medicine, Catholic University of Daegu, 33 Duryugongwon-ro 17-gil, Nam-gu, Daegu, Republic of Korea

[3]Korean Medicine (KM) Application Center, Korea Institute of Oriental Medicine, 70 Cheomdan-ro, Dong-gu, Daegu, 41062, Republic of Korea

[4]Research Institute of Biomedical Engineering and Department of Medicine, Catholic University of Daegu School of Medicine, 33 Duryugongwon-ro 17-gil, Nam-gu, Daegu, Republic of Korea

[5]Laboratory of Microbiology and Immunology, College of Pharmacy, Kangwon National University, 1 Kangwondaehak-gil, Chuncheon-si, Gangwon-do 24341, Republic of Korea

[6]Molecular and Cellular Glycobiology Unit, Department of Biological Sciences, SungKyunKwan University, 2066 Seobu-Ro, Suwon City, Kyunggi-Do 16419, Republic of Korea.

[7]Lipid Metabolism Project, Tokyo Metropolitan Institute of Medical Science, Tokyo 156-8506, Japan.

* Corresponding authors: Hyeun Wook Chang, Email: hwchang@yu.ac.kr; Cheol-Ho Kim, Email: chkimbio@skku.edu

Supplementary figures

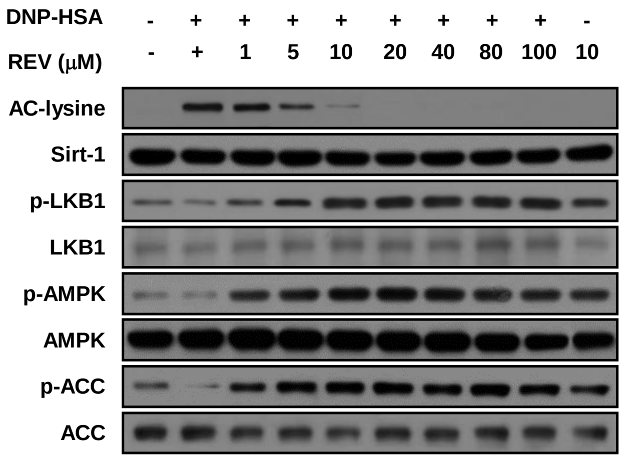

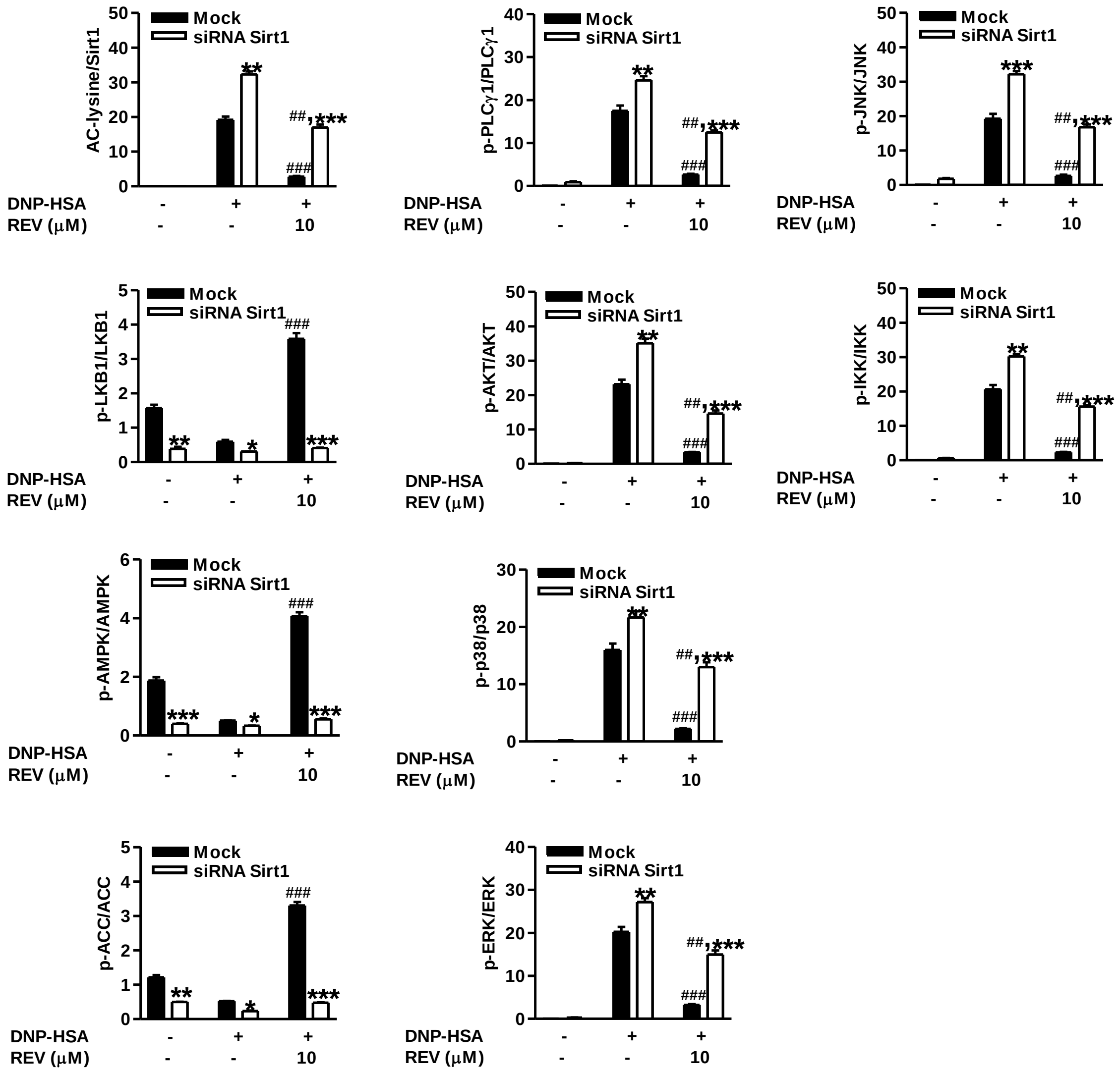
AC-lysine/Sirt1
p-PLCγ1/PLCγ1
p-JNK/JNK
p-LKB1/LKB1
p-AKT/AKT
p-IKK/IKK
p-AMPK/AMPK
p-p38/p38
p-ACC/ACC
p-ERK/ERK
Mock
siRNA Sirt1
DNP-HSA - + +
REV (μM) - - 10

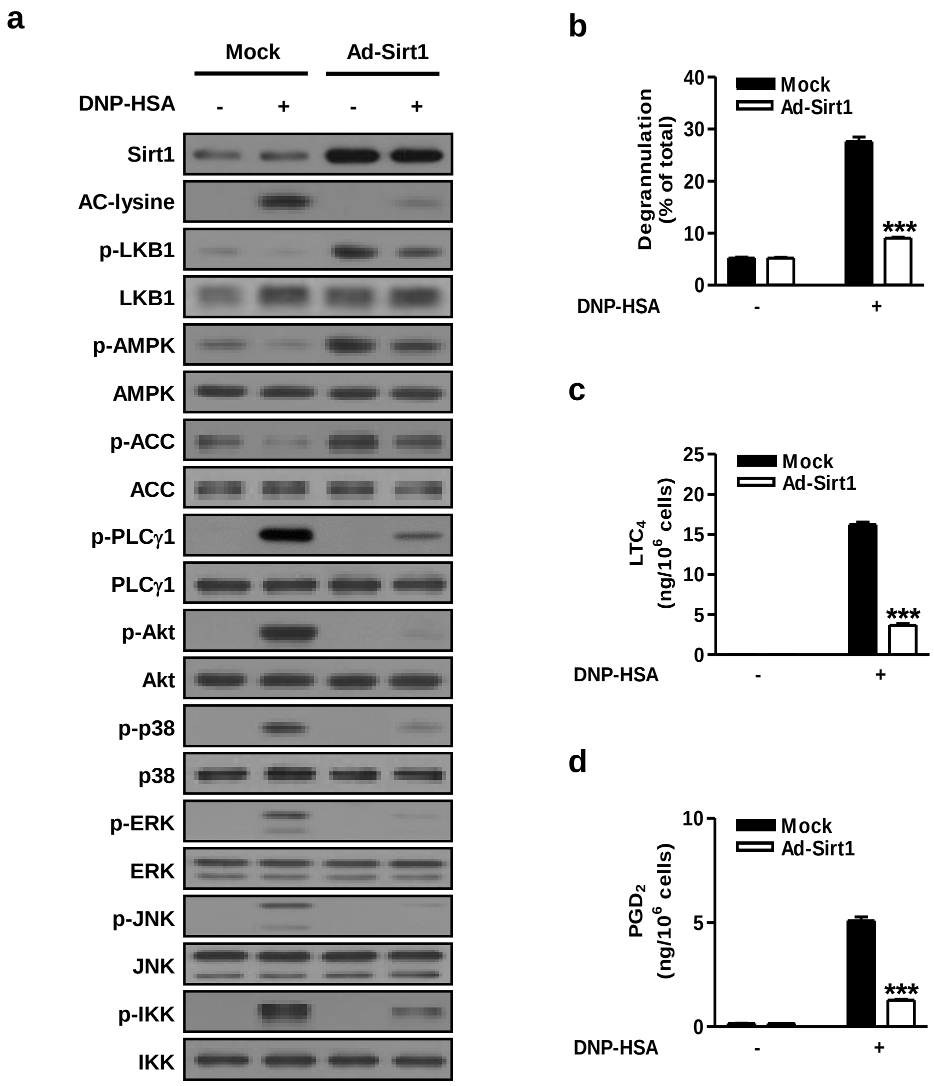

a
Mock
Ad-Sirt1
DNP-HSA
-
+
-
+
Sirt1
AC-lysine
p-LKB1
LKB1
p-AMPK
AMPK
p-ACC
ACC
p-PLCγ1
PLCγ1
p-Akt
Akt
p-p38
p38
p-ERK
ERK
p-JNK
JNK
p-IKK
IKK
b
Degrannulation (% of total)
Mock
Ad-Sirt1
***
DNP-HSA
c
LTC4 (ng/10^6 cells)
Mock
Ad-Sirt1
***
DNP-HSA
d
PGD2 (ng/10^6 cells)
Mock
Ad-Sirt1
***
DNP-HSA

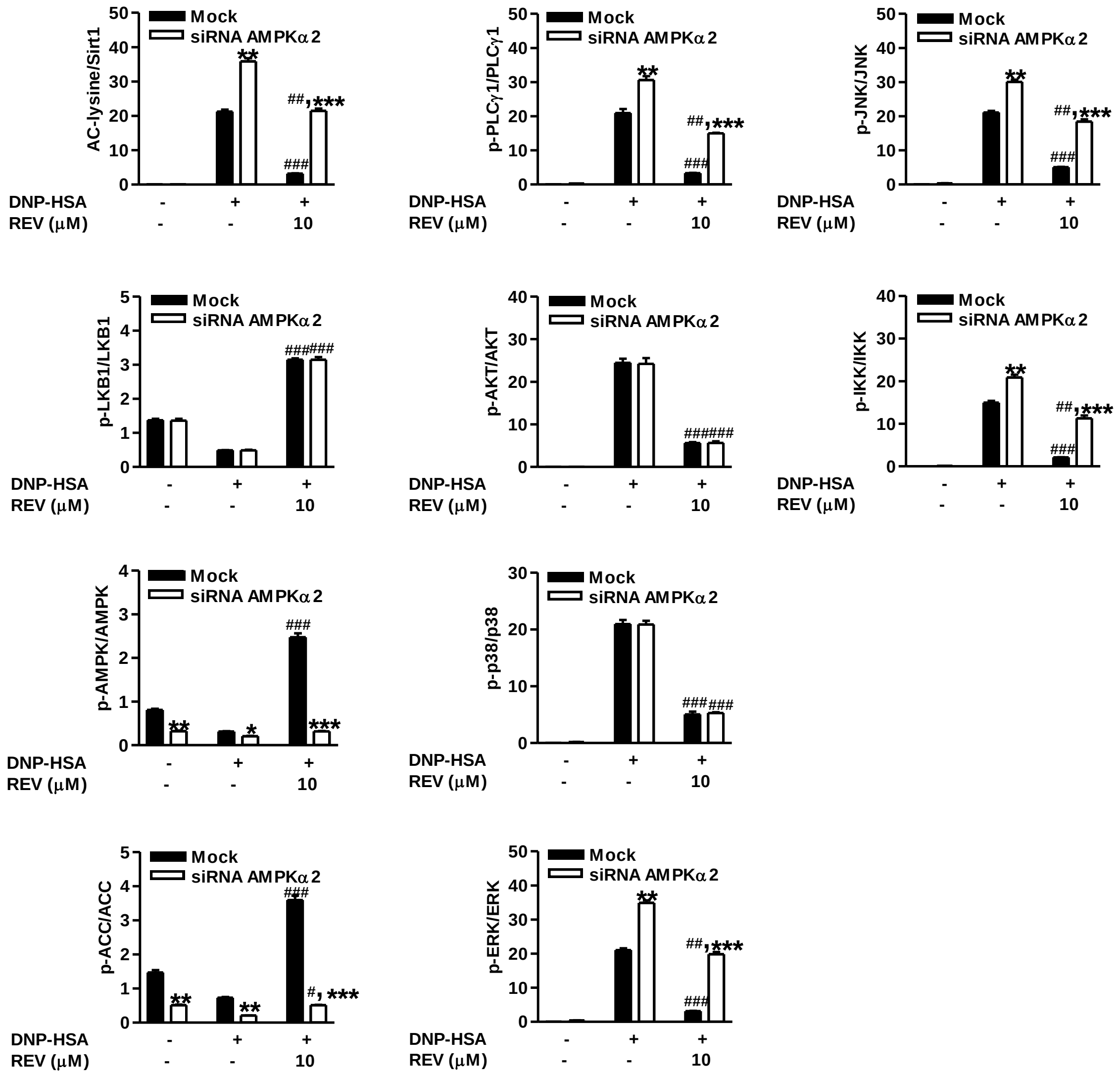
AC-lysine/Sirt1
p-PLCγ1/PLCγ1
p-JNK/JNK
p-LKB1/LKB1
p-AKT/AKT
p-IKK/IKK
p-AMPK/AMPK
p-p38/p38
p-ACC/ACC
p-ERK/ERK
Mock
siRNA AMPKα2
DNP-HSA
REV (μM)
- + +
- - 10

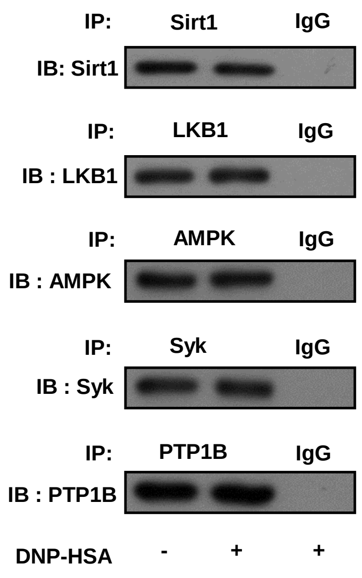
IP: Sirt1 IgG
IB: Sirt1
IP: LKB1 IgG
IB : LKB1
IP: AMPK IgG
IB : AMPK
IP: Syk IgG
IB : Syk
IP: PTP1B IgG
IB : PTP1B
DNP-HSA - + +

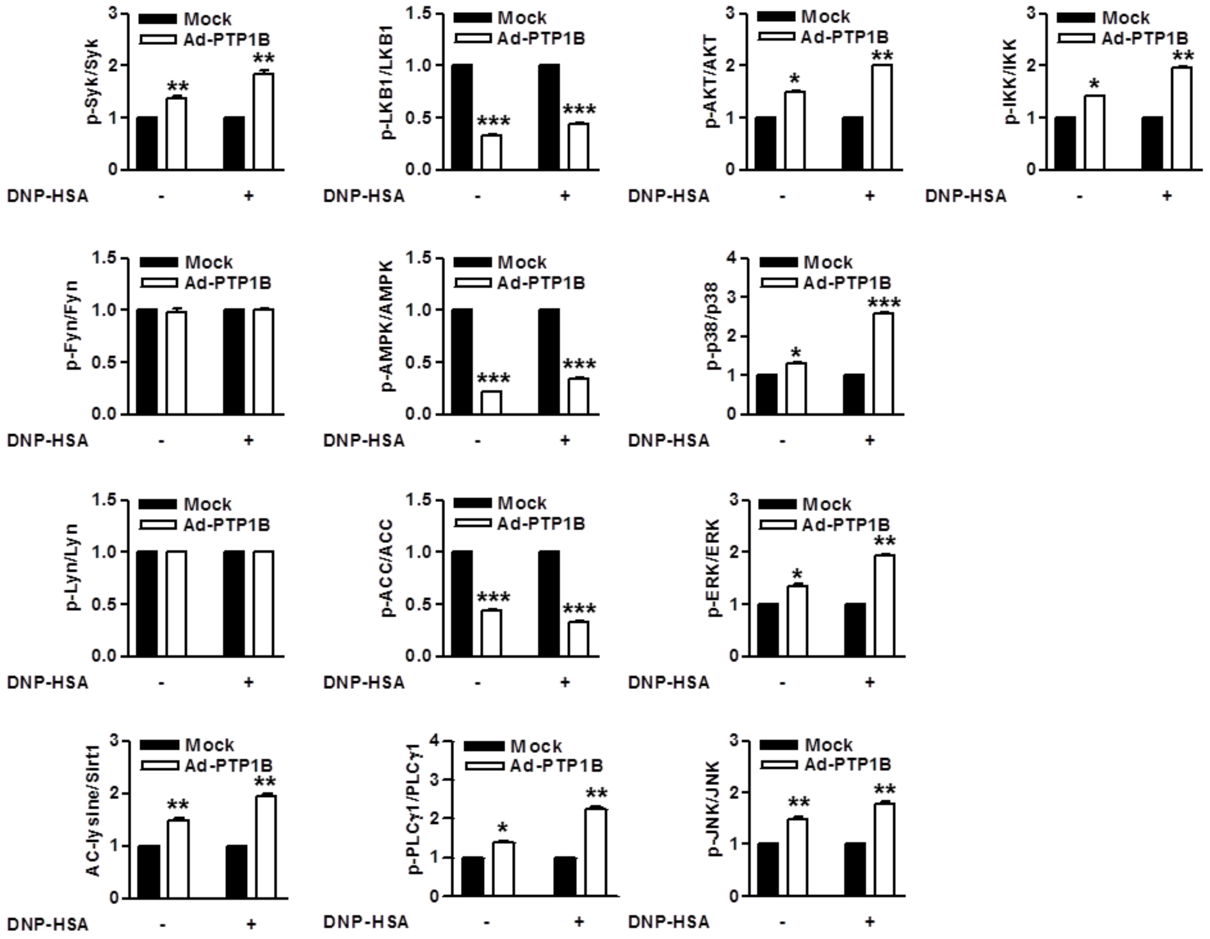
Mock
Ad-PTP1B
DNP-HSA
-
+
p-Syk/Syk
p-LKB1/LKB1
p-AKT/AKT
p-IKK/IKK
p-Fyn/Fyn
p-AMPK/AMPK
p-p38/p38
p-Lyn/Lyn
p-ACC/ACC
p-ERK/ERK
AC-lysine/Sirt1
p-PLCγ1/PLCγ1
p-JNK/JNK

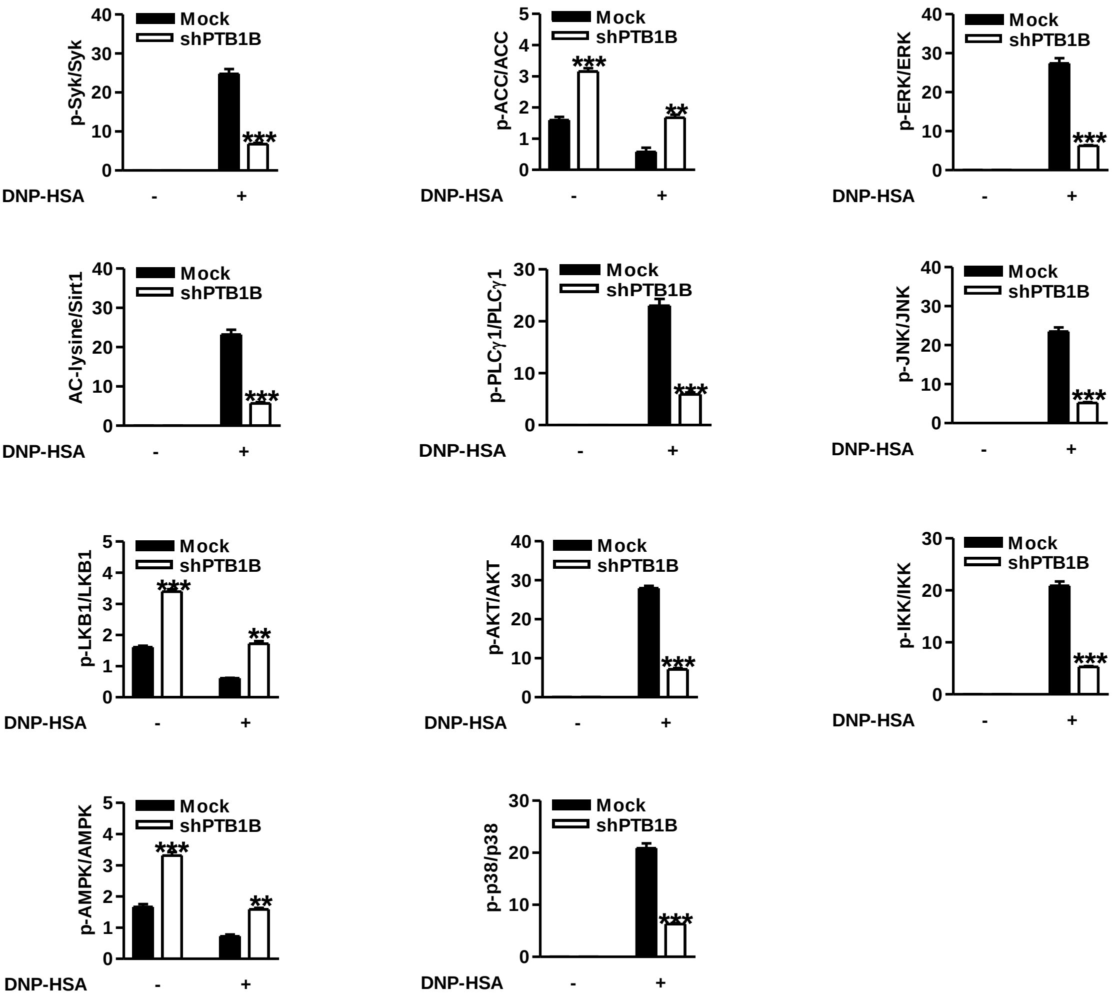
Mock
shPTB1B
p-Syk/Syk
p-ACC/ACC
p-ERK/ERK
AC-lysine/Sirt1
p-PLCγ1/PLCγ1
p-JNK/JNK
p-LKB1/LKB1
p-AKT/AKT
p-IKK/IKK
p-AMPK/AMPK
p-p38/p38
DNP-HSA
-
+
***
**

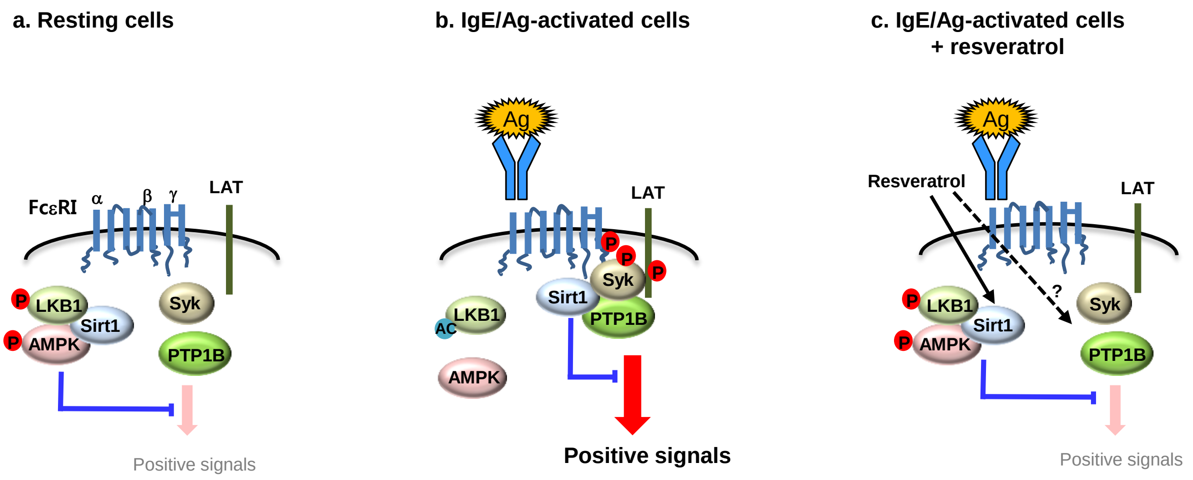
a. Resting cells
b. IgE/Ag-activated cells
c. IgE/Ag-activated cells
+ resveratrol
FcεRI
α
β
γ
LAT
P
LKB1
Sirt1
P
AMPK
Syk
PTP1B
Positive signals
Ag
LAT
P
P
Syk
P
Sirt1
PTP1B
AC
LKB1
AMPK
Positive signals
Ag
Resveratrol
LAT
?
P
LKB1
Sirt1
P
AMPK
Syk
PTP1B
Positive signals

Supplementary Fig. S1. Resveratrol dose-dependently suppresses the acetylation of lysine and phosphorylation of LKB1, AMPK and ACC in IgE/Ag-activated mast cells. IgE-sensitized BMMCs were treated with indicated concentrations of resveratrol (REV) or vehicle for 1 h and stimulated with Ag. Then, the acetylation of lysine (Ac-Lys) and the phosphorylation of LKB1, AMPK and ACC were analyzed by immunoblotting. The data is a representative of three independent experiments with different BMMCs.

Supplementary Fig. S2. Densitometric analysis of the effects of Sirt1 knockdown on acetylation or phosphorylation of signaling molecules in BMMCs. The relative ratios of intensity of acetylated- or phosphorylated- signaling molecules to their total proteins (panel Fig. 2a) were determined by scanning densitometry. The data from three independent experiments with different BMMCs are expressed as arbitrary units in bar graphs (*$P < 0.05$, **$P < 0.01$ and ***$P < 0.001$ *vs.* Mock in each treatment; $^{\#\#}P < 0.01$ and $^{\#\#\#}P < 0.001$ *vs.* DNP-HSA alone in mock or knockdown group).

Supplementary Fig. S3. Sirt1 overexpression alleviates FcεRI-mediated mast cell activation. BMMCs treated with adenovirus carrying Sirt1 (Ad-Sirt1) or control (mock) were stimulated with IgE/Ag. Acetylation or phosphorylation of signaling molecules (**a**) and releases of β-Hex (**b**), LTC_4 (**c**) and PGD_2 (**d**) were evaluated. The immuoblot data (**a**) is a representative of three independent experiments, and the values (**b-d**) indicate the means ± S.E.M. from three independent experiments with different BMMCs (***$P < 0.001$ *vs.* mock in each treatment).

Supplementary Fig. S4. Densitometric analysis of the effects of AMPKα2 knockdown on

acetylation or phosphorylation of signaling molecules in BMMCs. The relative ratios of intensity of acetylated- or phosphorylated- signaling molecules to their total proteins (panel Fig 3a) were determined by scanning densitometry. The data from three independent experiments with different BMMCs are expressed as arbitrary units in bar graphs (**P* < 0.05, ***P* < 0.01 and ****P* < 0.001 *vs.* Mock in each treatment; $^{\#\#}P < 0.01$ and $^{\#\#\#}P < 0.001$ *vs.* DNP-HSA alone in mock or knockdown group).

Supplementary Fig. S5. Validation of Immunoprecipitation of Sirt1, LKB1, AMPK, Syk and PTP1B. BMMCs were sensitized with IgE and then stimulated with or without DNP-HSA for 15 min. Cell lysates were subjected to immunoprecipitation using anti-Sirt1, -LKB1, -AMPK, Syk, or -PTP1B antibody, followed by immunoblotting using specific antibodies. Normal IgG was used as a negative control

Supplementary Fig. S6. Densitometric analysis of the effects of PTP1B overexpression on acetylation or phosphorylation of signaling molecules in BMMCs. The relative ratios of intensity of acetylated- or phosphorylated signaling molecules to their total proteins (Fig. 6a) were determined by scanning densitometry. The data from three independent experiments with different BMMCs are expressed as arbitrary units in bar graphs (**P* < 0.05 , ***P* < 0.01 and ****P* < 0.001 *vs.* Mock in each treatment).

Supplementary Fig. S7. Densitometric analysis of the effects of PTP1B knockdown on acetylation or phosphorylation of signaling molecules in BMMCs. The relative ratios of intensity of acetylated- or phosphorylated signaling molecules to their total proteins (Fig. 6e) were determined by scanning densitometry. The data from three independent experiments

with different BMMCs are expressed as arbitrary units in bar graphs (**$P < 0.01$ and ***$P < 0.001$ *vs.* Mock in each treatment).

Supplementary Fig. S8. A possible mechanism for the role of Sirt1 and PTP1B in the context of AMPK or Syk signaling in FcεRI-mediated mast cell activation. (**a**) In resting cells, the Sirt1/LKB1/AMPK complex maintains mast cell homeostasis by putting a brake on positive signal constitutively. (**b**) In activated cells, the PTPB1/Syk pathway activates positive signals. Sirt1 is dissociated from LKB1/AMPK and in turn complexed with PTP1B/Syk to prevent over-activation. (**c**) Resveratrol facilitates the formation of the Sirt1/LKB1/AMPK complex and the dissociation of the Sirt1/PTP1B/Syk complex, thereby sequestering positive signals. Resveratrol also acts through a Sirt1-independent process to inhibit PTP1B/Syk, whose mechanism remains unknown.
